# Role of LKB1 in lung cancer development

**DOI:** 10.1038/sj.bjc.6604515

**Published:** 2008-08-19

**Authors:** L Makowski, D N Hayes

**Affiliations:** 1 Division of Endocrinology, Department of Medicine, Sarah W Stedman Nutrition and Metabolism Center, Duke University Medical Center, Metabolism, and Nutrition, Durham, NC, USA; 2 Division of Medical Oncology, Department of Internal Medicine, Lineberger Comprehensive Cancer Center, University of North Carolina, Chapel Hill, NC, USA

**Keywords:** LKB1, STK11, Peutz-Jeghers syndrome, review, lung cancer, heritable tumour syndromes

## Abstract

Three phenotypically related genetic syndromes and their lesions (*LKB1, PTEN, and TSC1/2*) are identified as frequently altered in lung cancer. *LKB1*, a kinase inactivated in 30% of lung cancers, is discussed in this review. Loss of *LKB1* regulation often coincident with KRAS activation allows for unchecked growth and the metabolic capacity to accommodate the proliferation.

## Heritable tumour syndromes and lung cancer

Heritable tumour syndromes have provided invaluable insights into human disease. These focal perversions of normal growth and development shine as a beacon on particular genes and pathways in a manner, which, once deciphered, can impact far beyond the heritable syndrome itself. Just as a jigsaw puzzle assembled in parts suddenly comes together when a key piece is recognised, the genetic syndrome can bring together vastly disparate biologic knowledge to yield a bit of clarity where none existed before. Although the field of lung cancer has lacked such a fortuitous discovery to date, a series of recent events appears likely to open a new era in our understanding of the disease. Three genetic syndromes, Peutz–Jeghers syndrome (PJS), Cowden's disease (CD), and tuberous sclerosis (TS), all sharing parallel phenotypes primarily of non-malignant and non-pulmonary disease, have crystallised a common pathway, which is activated in a significant proportion of all human lung cancers. Key pieces have been identified and joined, such as nutrient metabolism, proliferative signalling, and anti-apoptosis pathways. The puzzle of lung tumour genesis, invasion, and metastasis is somewhat more complete.

## *LKB1* and PJS

The central figure in the current discussion is the gene *LKB1*. Its discovery as well as an appreciation of its biology is dominated by its causative role in the autosomal-dominant inherited PJS. First described in the 1920s by Johannes Peutz, an association with cancer was made by Harold Jeghers in the 1940s (Peutz, 1921; Jeghers *et al*, 1949). The syndrome includes an overgrowth of differentiated tissues called hamartomas, primarily polyps in the gastrointestinal tract (GI), as well as abnormal pigmentation of the mucous membranes and skin. Validating Dr Jegher's observations, large cohorts have clarified the risk and spectrum of malignancies in PJS, including a cumulative cancer risk of 60% by the age 60 years (Hearle *et al*, 2006). Of primary concern for PJS patients are tumours of GI origin, although breast, gynaecologic, pancreas, and lung have been reported.

The pattern of inheritance of PJS, as well as the high frequency of cancer in affected, indicated that a tumour suppressor gene was likely the causative agent. In 1997, Hemminki *et al* (1997) identified the chromosome region 19p13.3 as the relevant cytoband. A year later a single gene called serine/threonine kinase 11 (*STK11*) was identified as the culprit (Hemminki *et al*, 1998; Jenne *et al*, 1998). The transcript for *STK11* had previously been reported in an unmapped fashion by the more commonly used gene alias *LKB1*. The identification of a tumour suppressor and its genomic coordinates inspired a flurry of tumour resequencing surveys of *LKB1* in a range of tissues. Table 1 reviews a sample of the largest of these reports in the area of lung cancer, and includes associated epigenetic findings. Although infrequently mutated in spontaneous tumours, *LKB1* point mutations were seen in a pattern mirroring those seen in association with PJS, including cervix, gastrointestinal, and pancreas (Avizienyte *et al*, 1999; Sanchez-Cespedes, 2007). A range of atypical PJS tumours was also identified, including prostate and melanoma. Surprisingly, the highest mutation rate, hovering around 25% in multiple studies, was in lung adenocarcinomas, an established but uncommon PJS-associated tumour (Virmani *et al*, 1998; Sanchez-Cespedes *et al*, 2001) (Table 1).

## *LKB1* loss in cancer

The *LKB1* locus had been previously indicated as important in lung cancer, although admittedly without the laser focus of a known genomic syndrome and target gene. Indeed, 19p13.3 has been repeatedly indicated for its high frequency of loss of heterozygosity (LOH), often above 50%, in lung cancer by chromosomal analysis (Sobottka *et al*, 2000). Interestingly, the frequency of LOH for many tumours, including lung cancer, continues to be significantly higher than the reported rate of point mutations. The discrepancy between the reported rate of mutation and LOH raises a number of questions, including the accuracy of the reported mutation studies, the potential for other mechanisms of gene silencing (such as, promoter methylation or other epigenetic event), or the possibility that a second gene target lies in the region. In fact, careful attention to resequencing is crucial, and techniques that detect large homozygous deletions are required to fully characterize tumour *LKB1* loss (Ji *et al*, 2007). Yet, thorough sequencing alone does not fully account for the LOH mutation frequency discrepancy, prompting investigators to pursue other mechanisms of gene silencing. Promoter methylation accounts for a small fraction of repressed *LKB1* expression cases, but this is unlikely a major contributing mechanism (Esteller *et al*, 2000; Trojan *et al*, 2000). Other mechanisms of gene silencing, including microRNA, might contribute to some of the gap but these remain under investigation. Perhaps, the most interesting possibility is that *LKB1* haploinsufficiency itself could be tumorigenic, as we have recently shown in animal models (Ji *et al*, 2007). At this time, however, we cannot exclude the possibility that there is another gene target in the 19p13.3 region that could account for additional cases of LOH, such as *BRG1* (Medina *et al*, 2008). In contrast to the challenges of detecting functional *LKB1* gene dosage described above, two authors have reported that immunohistochemical assessment of LKB1 protein status as a straightforward proxy for both biallelic loss and promoter methylation (Ghaffar *et al*, 2003; Fenton *et al*, 2006). Taken together, information on genetic status of *LKB1* garnered through examining PJS patients and screening multiple tumour types has pointed to LKB1 as an important mediator in the development of cancer.

Frequent LOH at 19p13.3 points only indiscriminately to the importance of the *LKB1* locus in lung cancer, as many regions have chromosomal alterations in this disease. Similarly, the dramatic clinical histories of a minority of PJS patients include lung cancer, but not at such a high rate as to suggest the prevalence of *LKB1* alteration in this tumour (Hirano *et al*, 2002; Estrada Trigueros *et al*, 2005; von Herbay *et al*, 2005). Yet, cancers in a few young patients with PJS, indeed, suggest a role as a potential early carcinogenic event. Ghaffar *et al* (2003) have bolstered this possibility by detecting frequent *LKB1* loss in the progression of the premalignant lesion atypical adenomatous hyperplasia (AAH) towards frank invasive. Developmentally, LKB1 is expressed in foetal lung and ubiquitously expressed in adult bronchial mucosa, underscoring its importance in normal lung physiology and development (Luukko *et al*, 1999). Step by step, pieces of a puzzle assemble to document both the importance of *LKB1* to the normal lung and its high frequency of aberrancy in cancer. Yet, the reader might still rightfully wonder why does a gene that primarily causes genetic disease of the gut appear to be mutated so early and often in sporadic cases of lung cancer?

## LKB1 expression, regulation, and action

Like individual pieces of a jigsaw puzzle, the various facets of *LKB1* need further consideration before they reveal a coherent picture. The functional domains of *LKB1's* 10 exons (9 coding) include a central catalytic domain, nuclear localisation signal, putative cytoplasmic retention signal, and a prenylation motif (Alessi *et al*, 2006). Localised to the nucleus, it is transported to the cytosol and activated by binding two associated proteins STE20-related adaptor (STRAD), a catalytically inactive kinase-like protein, and an armadillo repeat-containing protein named mouse protein 25 (MO25). The complex is 100-fold more active as a kinase than LKB1 alone. LKB1 also associates with a chaperone complex consisting of HSP90 and Cdc37, which likely aids in stabilisation of LKB1 protein and assembly of the STRAD–MO25 complex. LKB1 is phosphorylated on several sites, either activating or inhibiting, by several kinases, including p90 ribosomal S6 kinase (RSK), protein kinase A (PKA), or ataxia-telengiectasia-mutated (ATM) kinase. Despite the apparently rich regulatory aspects of LKB1, however, no mutation hot spots have emerged.

**Figure 1:**
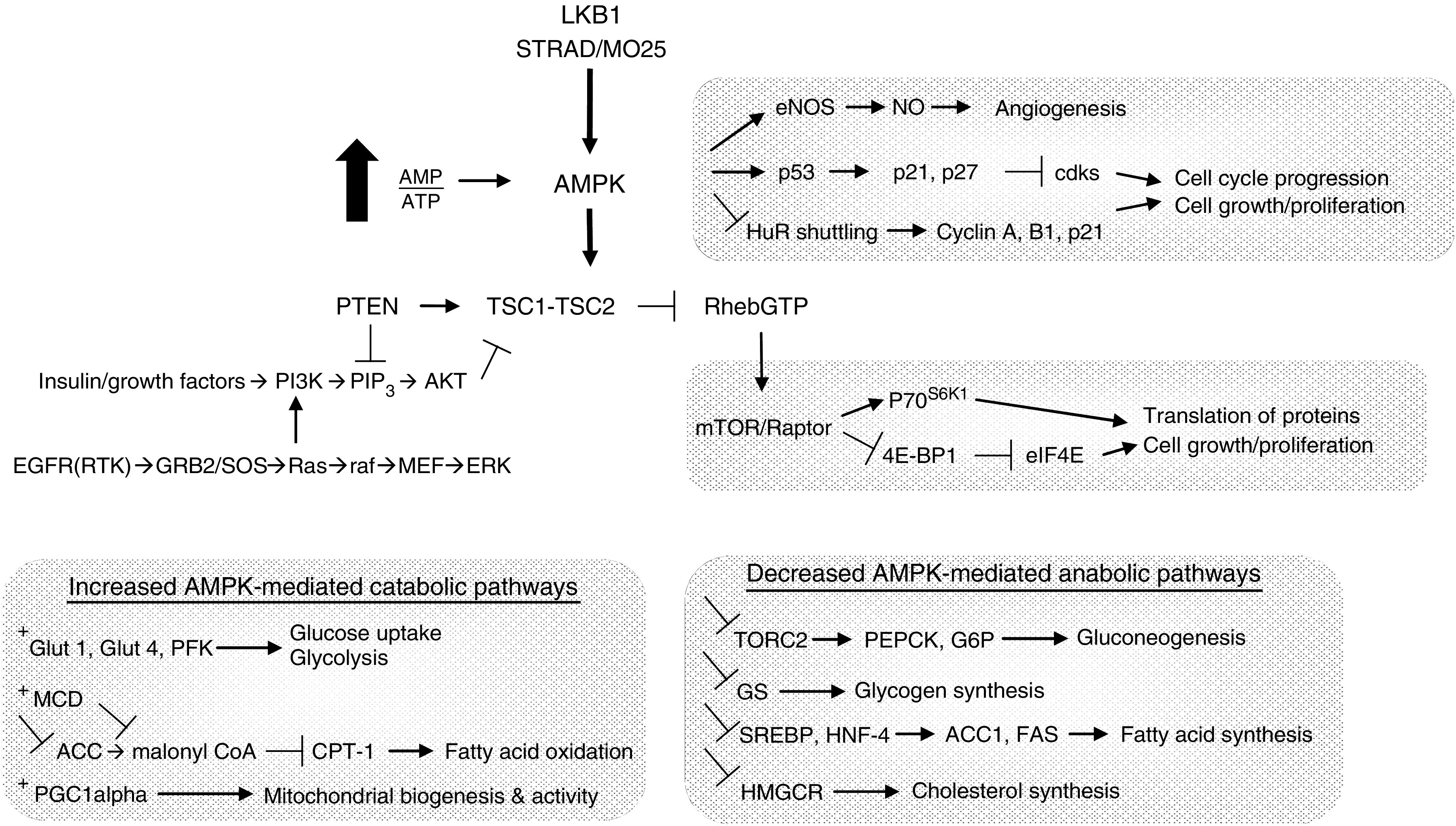
Energy sentinels in proliferation: LKB1 and AMPK. LKB1 is activated by binding STRAD and MO25. AMPK is a master regulator of metabolism, which is activated by LKB1 phosphorylation when the AMP/ATP ratio is high. AMPK drives catabolic pathways such as glucose uptake, glycolysis, mitochondrial proliferation, and fatty acid oxidation to generate ATP in times of stress. It also blunts ATP-consuming anabolic pathways including fatty acid, cholesterol, glycogen, and protein synthesis. The latter role of inhibiting protein synthesis through TSC1/2 and mTOR underlie AMPK's role in cell growth and proliferation. However, LKB1–AMPK regulation of fuel availability and the metabolic status is also central to proliferation. In fact, the LKB1 signal that cells should halt division when sufficient nutrients are not present overrides growth factor signalling through EGFR, for example. In sum, LKB1 and AMPK act together to sense energy stress to provide cells with nutrients for rapid energy production, while limiting growth. The sentinel activity of LKB1 and AMPK is lost in cancer, which allows unlimited proliferation despite metabolic cues to the contrary.

LKB1 functions as a tumour suppressor that regulates cell polarity, differentiation, and metastasis as well as responds to energy status to regulate cell metabolism (Figure 1) (see Alessi *et al*, 2006; and references within). One function of LKB1 directly linked to lung cancer is the phosphorylation, ubiquitination, and the degradation of polyomavirus enhancer activator 3 (PEA3). Mutant LKB1 increases the stability of PEA3, which, in turn, increases transcription of genes involved in metastasis, including *COX-2*, *MMP-9*, and *MMP-14* (Upadhyay *et al*, 2006; Cowden Dahl *et al*, 2007). Interestingly, COX-2 is commonly elevated in many disease states, including PJS and lung cancer (Achiwa *et al*, 1999; Rossi *et al*, 2002; McGarrity *et al*, 2003). Indeed, COX-2 inhibition decreased polyps in a small study of PJS patients and a large study of patients with familial adenomatous polyposis (Udd *et al*, 2004; Bertagnolli *et al*, 2006). Finally, increased COX-2 is associated with decreased survival in patients with lung adenocarcinoma (Achiwa *et al*, 1999). Although LKB1 clearly regulates proteins such as PEA3, current understanding is that its primary action is through the target 5′-AMP-activated protein kinase (AMPK) (Towler and Hardie, 2007). AMPK is a master regulator of metabolism that orchestrates efficient energy production with minimal waste in times of energy stress. It is a serine/threonine kinase activated by metabolic signals and stressors, including exercise, starvation, hypoxia, and ischaemia. In short, consumption of ATP, decreased ATP production, and/or increased concentration of AMP activate AMPK to alter numerous metabolic and proliferative pathways (Figure 1). Downstream of LKB1, AMPK tightly regulates the metabolic status of cells. For example, when carbon sources are scarce, AMPK ensures ATP generation by activating catabolic pathways such as glycolysis and fatty acid oxidation, as well as upregulates mitochondrial biogenesis. In parallel, it switches off ATP-consuming anabolic pathways, including fatty acid, cholesterol, glycogen, and protein synthesis. Of relevance to this review, AMP binding to AMPK only causes a fivefold increase in activity, while the AMP-induced conformational change in AMPK primes it as a substrate for LKB1, allowing LKB1 phosphorylation to induce activation by 100-fold (Alessi *et al*, 2006). Therefore, AMPK action and LKB1 activity are tightly coordinated to sense energy stress and attempt to provide cells with fuel for rapid energy production, while limiting growth and energy loss when nutrients are scarce.

## *LKB1* proliferation, metastasis, and metabolism

A central target for AMPK's control of proliferation is the mammalian target of rapamycin (mTOR) kinase, which regulates numerous downstream targets, such as amino acid transporters, VEGF, p70 ribosomal protein S6 kinase 1 (S6K), and eukaryotic initiation factor 4E-binding protein 1 (4E-BP1) pathways, among others, to increase the translation of proteins and cell growth (Figure 1). In fact, AMPK acts on mTOR through phosphorylating and activating tumour suppressor tuberous sclerosis complex-2 (TSC2), an upstream negative regulator of mTOR. AMPK activation also blocks cell cycle progression from G1 to S phase through phosphorylation and accumulation of the tumour suppressor p53 and the downstream cyclin-dependent kinase inhibitors p21^WAF1/CIP1^ and p27. Other cell cycle regulation occurs through the alteration of cytoplasmic/nuclear ratios of RNA-binding protein human antigen R (HuR), thereby reducing the ability to stabilise mRNAs encoding cyclins (Wang *et al*, 2002). Finally, hypoxia-induced AMPK activation of endothelial NOS (eNOS) drives angiogenesis. The relative importance of each mechanism discussed remains to be determined. In sum, *LKB1* inactivation in cancer releases the AMPK-mediated breaks on energy waste and permits proliferation. The capacity to sense a nutrient deficit is a function of healthy cells that is lost in proliferating cells, which highlights a distinctly separate but parallel piece of the cancer puzzle.

## Hamartomatous syndromes, *mTOR*, and lung cancer

Decoding the regulatory networks of LKB1, particularly an interaction with the AMPK–mTOR axis, crystallises a link between a genetic disease with little pulmonary implications and a gene frequently altered in lung cancer. Clearly, *mTOR* is a lung cancer target; yet, the jumble of alternate pathways raises doubts as to whether this is the primary mechanism (Figure 1). Quite remarkably, upstream of the mTOR pathway, however, is the invocation of three genes and two autosomal-dominant inherited syndromes with phenotypes remarkably similar to PJS, including *PTEN* (CD) and *TSC1/TSC2* (tuberous sclerosis, TS). Like PJS, both CD and TS manifest themselves as primary hamartomatous diseases. Patients with CD demonstrate hamartomas of the skin, mucous membranes, breast, and thyroid, with 85% of patients showing *PTEN* germline mutation. Like PJS, an increased risk of tumours is seen, although again lung cancer is not prominent. The PTEN phosphatase controls the levels of PIP_3_ induced by growth factor activation of PI3 kinase, and acts to negatively regulate mTOR activity. Patients with TS do not have malignancy as their primary concern, but instead hamartomas of the skin, brain, kidney, skin, lungs, and other tissues (Crino *et al*, 2006). As in CD, the TS complex acts as a negative regulator of mTOR. In summary, the related phenotypes of PJS, CD, and TS are joined by shared regulation of the mTOR signalling cascade.

Consideration of these syndromes begs the question, is there a broader lung cancer malignant phenotype equivalent to that shared by germline PJS, CD, and TS? In fact, mutations of *PTEN* have been reported in many tumours, including 4–8% of non-small-cell lung cancers (NSCLC) (Forbes *et al*, 2006). PTEN protein expression is lost in more than 25% of NSCLC tumours with evidence of epigenetic silencing at work as well (Luukko *et al*, 1999; Marsit *et al*, 2005). Additionally, PTEN is co-expressed with LKB1 in foetal lung development. Similarly, both the *TSC1* locus (9q34) and the *TSC2* locus (16p) are frequent targets of LOH in both lung adenocarcinoma and the pre-invasive lung lesion AAH (Takamochi *et al*, 2001, 2004). Although the frequency with which these events occur in concert remains unknown, the conclusion must be that targets coalescing on the mTOR pathway defined in large part by dissecting the disease of germline mutations of *LKB1*, *PTEN*, and *TSC1/2* appear to be frequently altered in lung cancer, suggesting a broad and important contribution of this specific set of interacting proteins to the disease.

## Turning to animal models: *LKB1* and *KRAS*

Recent years have seen progress similar to that we document for *LKB1* in a number of other lung cancer genes and cancer networks, such as *EGFR*, *KRAS*, *p53*, and *CDKN2A* (including both transcripts: p16^Ink4a^ and INK4a/ARF). As we suggest above, it is expected that the alteration of only one gene in a pathway may be sufficient for tumorigenesis. As such, an understanding of the relationships between aberrations may help clarify the minimal set of events necessary to cause cancer. To this end, we documented several interactions between LKB1 and other genes commonly altered in lung cancer in experimental animal models (Ji *et al*, 2007). As predicted from the natural human experiment of PJS, mice deficient in *LKB1* do not get lung tumours. The alteration of other lung cancer genes in combination with *LKB1*, however, did produce tumours of a striking phenotype. First, we observed that homozygous loss of *LKB1* in combination with *KRAS* resulted in an aggressive tumour phenotype with high tumour multiplicity, short survival, and frequent metastases well above those of *KRAS* alone. The *KRAS/LKB1* double knockout was in fact the most aggressive phenotype of all tumours considered in the study, and had an additional feature that had not previously been reported in a mouse model: squamous cell carcinoma histology. Additionally, it was noted that even hemizygous loss of *LKB1* in combination with *KRAS* resulted in a more aggressive phenotype than in *KRAS* mutation alone.

The clinical relevance of *LKB1* alteration remains an area of active investigation, although preliminary results appear likely to confirm findings of the animal models, including the frequent co-mutation of *LKB1* inactivation and activating mutations of *KRAS* (Table 1). As a mirror opposite to the established positive association of *EGFR* gene mutation with females and nonsmokers, we detect a positive association of *LKB1* inactivation with both smoking and male gender. Other interesting reports, including prevalence of brain metastasis, associations with poorly differentiated tumours, the importance of the hemizygous *LKB1* state, and apparent independence with *p53* mutation, remain to be confirmed. Certainly, we hope for clinical associations of this type on which to base important patient management decisions. In broader terms, one has to note that many of the tumours associated with PJS, including GI and gynaecologic malignancies are also those where *KRAS* is a frequent target of mutation. The question remains, is the *LKB1–KRAS* connection part of a broad phenotype of interconnecting cancer pathways? We propose that perhaps *KRAS* activation and *LKB1* loss permit liberation from cell cycle and energy status checkpoints, respectively, which allow for a metabolic advantage.

## *mTOR* and *KRAS* in the clinic

Paramount in the *LKB1* story is the focus now thrust upon very specific cancer pathways, several of which have attractive therapeutic targets. At the top of the list is the mTOR pathway where three Food and Drug Administration (FDA)-approved inhibitors are currently available, such as rapamycin, everolimus, and temsirolimus, with more in development, including the recently described deforolimus (Cohen, 2008). Inhibitors of mTOR have already been investigated in lung cancer, with clear evidence of anticancer activity both as single agents and in combination with cytotoxic chemotherapy and radiation (Milton *et al*, 2007; Sarkaria *et al*, 2007; Mita *et al*, 2008). Interestingly, a recent report in the *New England Journal of Medicine* documented a striking therapeutic effect of mTOR-targeted therapy by sirolimus to angiomyolipomas, which are lesions attributable to TSC dysfunction in patients with TS (Bissler *et al*, 2008). Patients with angiomyolipomas, including the pulmonary form lymphangioleiomyomatosis, almost uniformly experienced reduction in the size of their tumours with this therapy. The efficacy of mTOR-targeted therapy in this benign tumour of the lung, marked by one aspect of mTOR activation, certainly is of interest due to its parallel biology in lung cancer.

Although activity in these early studies has been documented, there is little evidence that this will be of a broader spectrum than for other classes of lung cancer therapeutics. Therefore, there is an incentive to define biomarkers of response to therapy, such as mutation status or, perhaps, more encompassing markers such as those indicating AMPK activity. There is increasing evidence that broad patterns of tumour behaviour can be captured as general phenotypes using profiling techniques such as gene expression arrays. Specifically, the squamoid gene expression subtype of lung adenocarcinoma is known to have higher rates of *KRAS* mutation and demonstrates gene expression correlated with *LKB1* inactivation (Hayes *et al*, 2006). It is also interesting to note that as *LKB1* mutation appears to pair with *KRAS* activation, specific combinations of therapy targeting parallel pathways might be appropriate. Indeed, initial work on this similar line has begun, including targeting downstream elements of RAS signalling (MEK) and mTOR simultaneously (Legrier *et al*, 2007).

## Conclusion

Three phenotypically related genetic syndromes, including PJS, CD, and TS, are united by careful alignment of their mechanisms acting through the mTOR pathway. The lesions responsible for the syndromes, *LKB1*, *PTEN*, and *TSC1/2*, are identified as frequently altered in lung cancer, suggesting that they comprise elements of a common lung caner phenotype. Taken together, when the phenotype of *LKB1* mutation is examined in the setting of known alterations of lung cancer, an interesting association of *KRAS* dependence appears, with specific clinical and treatment potential. Like all good puzzles, the pieces are starting to fall into place.

## Figures and Tables

**Table 1:** Summary results of studies reporting LKB1 mutation and other related events in lung cancer

**Reference**	**Year**	**Finding**	**Method**	**Comments/observations**
Virmani *et al* (1998)	1998	10/12 LOH in NSCLC cell lines and 3/9 in SmCC cell lines in 19p13.2	Allelotyping by PCR	Lung cancer cell lines used to define common, specific known, and novel deletions in NSCLC and SmCC cell lines
Sobottka *et al* (2000)	2000	7/12 (58%) patients with LOH at 19p in brain metastases of lung cancer patients, whereas glioma *LKB1* LOH was <20%	Microsatellite PCR analysis and genomic sequencing	None of brain metastases showed *LKB1* mutations by direct genomic sequencing
Sanchez-Cespedes *et al* (2002)	2002	5/20 of primary AC, 0/12 primary SCC; and 2/9 of lung cancer cell lines with mutation of *LKB1*	Microsatellite PCR, direct sequencing, long-range PCR	0/12 mutations in SCC
		1/20 tumor with *LKB1* promoter hypermethylation	Methylation-specific PCR	
				
Ghaffar *et al* (2003)	2003	9/35 loss of *LKB1* protein in AC, 10/96 loss in AAH (7/33 loss in high-grade AAH; 3/63 loss in low grade; 9/35 invasive tumors)	Immunohistochemistry	LKB1 protein loss from AAH correlated with severe dysplasia, suggesting loss as essential to transition from pre- to malignant tumor. Also showed strong positive association between biallelic gene inactivation and negative protein expression
Fernandez *et al* (2004)	2004	5/19 lung AD with point mutations of *LKB1*	Direct sequencing, long-range PCR	Frequent observation of concurrent *KRAS* (4/11) and *p53* (6/11) in *LKB1* mutant samples. Performed microarray analysis with several genes dependent on *LKB1* status
		0/19 samples with promoter methylation of *LKB1*	Methylation-specific PCR	
		10/18 of samples with LOH at 19p	Fluorescent *in situ* hybridization	
				
Carretero *et al* (2004)	2004	6/11 AD contained *LKB1* deletion or truncating mutant, 0/11 SCC	Allelotyping by PCR and sequencing	Several AD lines with *LKB1* mutant also have *KRAS* mutant
Matsumoto *et al* (2007)	2007	Lung cancer cell lines with mutations in *LKB1*: 13/31 AD, 1/2 ADSCC, 3/11 SCC, 3/7 LCC, and 1/19 SmCC	RT-PCR, sequencing and genomic sequencing	Strong positive correlation with *KRAS* status, not correlation with *p53* status or *EGFR* mutation status
		8/70 lung cancer cell lines demonstrated shortened *LKB1* transcripts, 9/70 demonstrated absent *LKB1* transcript		Two cell lines with absent gene expression did not demonstrate mutation, suggesting alternate gene silencing method. The 9/70 with loss of *LKB1* had deletions in promoter or exon 1
		Primary tumors: 1/106 stage 1 tumor with *LKB1* mutation, 3/24 stages I–III. 7/91 male smokers, 0/64 female non-smokers		Small numbers limit interpretation for *KRAS* and *EGFR* associations, appears to be associated with poorly differentiated tumors
		3/25 brain metastases of lung cancer patients with *LKB1* mutation		
				
Ji *et al* (2007)	2007	Primary tumors: 19/80 AD, 6/42 SCC, 1/7 LCC, and 1/4 ADSCC, respectively, with single copy alterations of *LKB1*; 8/80 AD, 2/42 SCC with homozygous mutations	Direct sequencing, multiplex ligation-dependent probe amplification	SCC, AD, SCC, LCC, and AD: frequent loss of a single copy of *LKB1*, concomitant *p53*, and *KRAS* mutations found frequently
Onozato *et al* (2007)	2007	Cell lines with mutations in *LKB1*: 3/8 AD, 1/6 SCC, 1/3 LCC, and 0/5 SmCC	Direct sequencing	Lung cancer cell lines examined
		3/100 primary tumors with *LKB1* mutations in Japanese cohort 3/33 male smokers	Direct sequencing	*LKB1* mutation rare in Japanese lung cancer population. All mutations were in male smokers with moderately or poorly differentiated tumors

ADSCC=adenosquamous carcinoma; AD=adenocarcinoma; AAH=atypical adenomatous hyperplasia; LCC=large cell carcinoma; LOH=loss of heterozygosity; NSCLC=non-small-cell lung cancer; PCR=polymerase chain reaction; SmCC=small cell carcinoma; SCC=squamous cell carcinoma.
